# Modulation of miRISC-Mediated Gene Silencing in Eukaryotes

**DOI:** 10.3389/fmolb.2022.832916

**Published:** 2022-02-14

**Authors:** Courtney F. Jungers, Sergej Djuranovic

**Affiliations:** Department of Cell Biology and Physiology, Washington University School of Medicine, St. Louis, MO, United States

**Keywords:** miRNA, miRISC, RNA binding protein, mRNA, miRISC activity, RBP binding models

## Abstract

Gene expression is regulated at multiple levels in eukaryotic cells. Regulation at the post-transcriptional level is modulated by various *trans*-acting factors that bind to specific sequences in the messenger RNA (mRNA). The binding of different *trans* factors influences various aspects of the mRNA such as degradation rate, translation efficiency, splicing, localization, etc. MicroRNAs (miRNAs) are short endogenous ncRNAs that combine with the Argonaute to form the microRNA-induced silencing complex (miRISC), which uses base-pair complementation to silence the target transcript. RNA-binding proteins (RBPs) contribute to post-transcriptional control by influencing the mRNA stability and translation upon binding to *cis*-elements within the mRNA transcript. RBPs have been shown to impact gene expression through influencing the miRISC biogenesis, composition, or miRISC-mRNA target interaction. While there is clear evidence that those interactions between RBPs, miRNAs, miRISC and target mRNAs influence the efficiency of miRISC-mediated gene silencing, the exact mechanism for most of them remains unclear. This review summarizes our current knowledge on gene expression regulation through interactions of miRNAs and RBPs.

## Introduction

The central dogma of biology follows the flow of genetic information; DNA is transcribed into RNA and RNA is translated into protein. Correct gene expression in a timely and quantitative way is essential for maintaining cellular homeostasis as dysregulated protein production can lead to various diseased states. RNA is not just a simple intermediate for conveying the genetic code, but it also regulates when, where, and how much protein will be produced. RNA has a complex, multistage lifecycle starting in the nucleus, where it is transcribed from DNA. After maturation, which includes co- and post-transcriptional processing, including 5′ capping, polyadenylation, and splicing, the mRNA is exported into the cytoplasm from the nucleus. In the cytoplasm ribosomes translate coding mRNAs into protein. From its initial transcription until its degradation, mRNAs are highly regulated at both a global and individual level. Individual mRNAs are regulated by various *trans*-acting factors that bind to specific *cis*-regulatory elements within the mRNA and influence the stability, localization, modifications, and translation of the information encoded in the mRNA (Gebauer and Hentze, 2004; Fukao et al., 2021).

Several classes of small noncoding RNAs (ncRNAs) have been shown to bind to specific sequences within mRNAs, mainly in the 3′ untranslated region (UTR), and influence the extent of expression of encoded genes. The three main classes of small ncRNAs, within 19–31 nucelotide length, are microRNAs (miRNA), small interfering RNAs (siRNAs), and piwi-interacting RNAs (piRNA). Each class of these small ncRNAs associates with distinct sets of effector proteins to carry out their function, both miRNAs and siRNAs associated with the Ago-clade, while piwi-RNAs associate with the PIWI-clade (Parker and Barford, 2006; Peters and Meister, 2007; Farazi et al., 2008; Thomson and Lin, 2009; Juliano et al., 2011). miRNAs and siRNAs are the most well understood and similar of the three; they are mostly derived from endogenous encoded double-stranded hairpin-shaped RNA. The main difference between miRNAs and siRNAs is their complementarity; siRNAs are derived from stem-loop with perfect complementarity while miRNAs contain imperfect complementarity. This review focuses on miRNAs, but siRNAs and piRNAs have been well-documented in recent reviews (Farazi et al., 2008; Carthew and Sontheimer, 2009; Thomson and Lin, 2009; Dana et al., 2017; Czech et al., 2018).

miRNAs are conserved, endogenous, short (19–22 nt) ncRNAs that combine with Argonaute (Ago) proteins to form the microRNA-induced silencing complex (miRISC). The miRISC uses imperfect base pair complementarity to bind to specific sequences found mostly in the 3′UTR of target mRNAs and repress the gene expression of that transcript (Siegel et al., 2011; Lin and Gregory, 2015; Duchaine and Fabian, 2019). While there were hints of miRNAs in the 1980’s in developmental and cell lineage screens (Horvitz and Sulston, 1980), the first miRNA, lin-4, was discovered in 1993 by the Ambros lab (Lee et al., 1993). Lin-4 was found to bind to the lin-14 mRNA and post-transcriptionally repress the expression of the Lin-14 protein. Lin-14 is necessary for proper timing of larval development in *Caenorhabditis elegans* (*C. elegans*) as loss-of-function lin-4 mutants revert to an early developmental stage later in their development. Seven years later the, second miRNA, let-7, was discovered (Reinhart et al., 2000). As let-7 was also found to be a heterochromatic switching factor, it was first thought that these short, hence the name micro, ncRNAs must target mRNAs that code for developmental genes. However, as more miRNAs continued to be discovered, their roles became more diverse, suggesting a much broader role in biological processes (Bartel, 2018). Over 60% of human protein-coding genes have been shown to be targeted by miRNAs (Friedman et al., 2008). As miRNAs modulate the expression of genes involved in cellular differentiation, division, growth, and apoptosis it comes to no surprise that the miRNAs themselves must be highly regulated to avoid diseased states (Siomi and Siomi, 2010). In addition to small ncRNAs, RNA-binding proteins (RBPs) are another class of *trans*-factors that bind to *cis* elements within mRNA transcripts. RBPs regulate many aspects of processes associated with RNAs; splicing, transcription, modification, localization, translation, and decay (Díaz-Muñoz and Turner, 2018; Hentze et al., 2018). RBPs have been found to greatly influence both miRNA biogenesis and miRNA-mediated gene silencing. This review focuses on miRNA-mediated gene silencing and investigates the influence of RBPs on the modulation of miRISC function (Gebauer et al., 2021).

## miRNA Biogenesis in Eukaryotes

**FIGURE 1:**
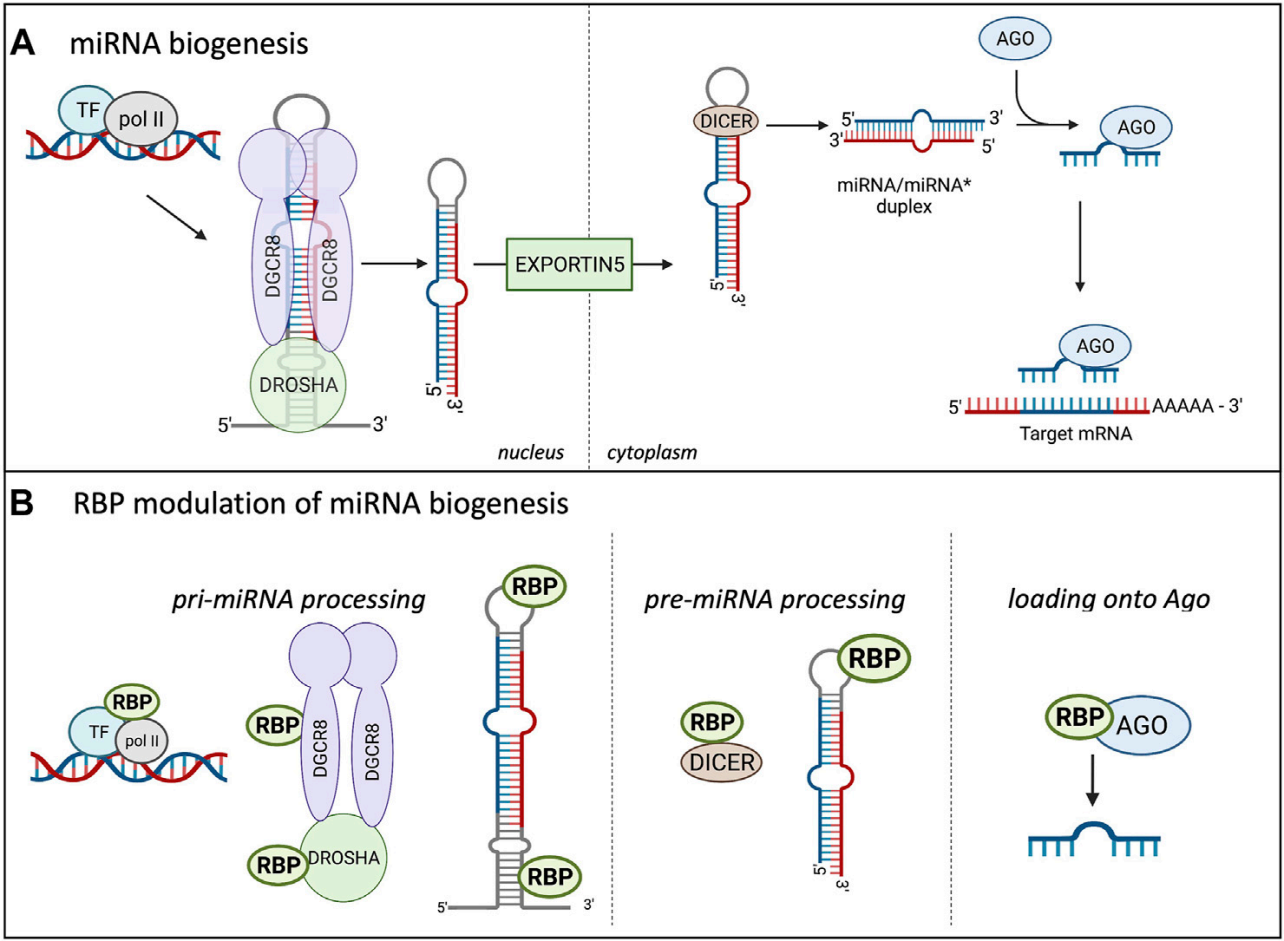
Canonical miRNA biogenesis in Eukaryotes and the influence of RBPs. **(A)** shows the miRNA biogenesis in eukaryotes. miRNAs are transcribed in the nucleus by RNA polymerase II (pol II), creating the pri-miRNA, two sequential cleavage reactions follow. The microprocessor consists of Drosha and DGCR8 and performs the first cleavage reaction in the nucleus, creating the pre-miRNA. The pre-miRNA is transported into the cytoplasm through Exportin5 where the second cleavage reaction occurs. Dicer cleaves the terminal loop of the pre-miRNA, creating the miRNA/miRNA* duplex. The miRNAs are incorporated into the Ago protein, forming the minimal effector RNA induced silencing complex (miRISC) and target mRNA sequences. **(B)** Highlights the modulation of miRNA biogenesis by RBPs. RBPs can bind to the promoter region of certain miRNAs and influence their transcription. RBPs modulate miRNA expression at the pri-miRNA processing level through binding to Drosha and enhance or repress the cleavage. RBPs can also bind to the terminal loop or other sequences in the pri- and pre-miRNAs to influence the cleavage reactions. Additionally, RBPs can bind to DICER and influence this cleavage reaction through modulating DICER expression and availability. Lastly, RBPs can bind to AGO and increase the miRNA loading onto the AGO, increasing the miRISC silencing.

As miRNAs play a major role in controlling protein abundance, the expression and number of miRNAs is highly important. As depicted in Figure 1A, prior to silencing their target transcripts, miRNAs are transcribed in the nucleus mostly by RNA polymerase II, forming the primary (pri) miRNA. The pri-miRNA is composed of a local stem loop containing 3 helical stems that are flanked by basal and apical junctions at both ends (Ha and Kim, 2014; Nguyen et al., 2015; Bartel, 2018; Duchaine and Fabian, 2019). Figure 1A shows how the pri-miRNA is processed in the nucleus to form the shorter pre-miRNA. The pre-miRNA is created through two sequential processing reactions; first, the pri-miRNA hairpin is recognized and cleaved from the transcript by the microprocessor, which comprises Drosha and the double-strand RNA (dsRNA) binding protein DGCR8 (DiGeorge Critical Regulator 8). DGCR8 helps provide the affinity for the microprocessor as it acts as a molecular anchor to position Drosha’s catalytic site at the desired distance from the stem flanking region (Bartel, 2004, 2018). DGCR8 interacts with the stem and apical portion of the stem-loop structure in the pri-miRNA and positions Drosha for cleavage (Nguyen et al., 2015; Figure 1A). Drosha, a member of the Rnase III family, localizes at the pri-miRNA basal junction and cleaves the stem-loop from the rest of the transcript, creating a pre-miRNA product ∼60–75 nucleotides long (Ha and Kim, 2014). There are multiple transcripts with the ability to fold backward and form hairpins, but not all are selected to become pri-miRNAs and enter the miRNA biogenesis pathway. The microprocessor is the gate-keeper of this process and seems to have a preference for pri-miRNAs containing hairpins with a stem that is ∼35 base pairs long, an unstructured apical loop that is over 10 nucleotides long, single-stranded sequences flanking the hairpin, and there are 4 sequence motifs at sites correlating to the position of the microprocessor. These sequence motifs include a basal UG motif, an apical UGU motif, a CNNC flanking motif, and a mismatched GHG motif with 6 nucleotides of the basal stem (Fang and Bartel, 2020; Shang et al., 2020). However, not all pri-miRNAs are optimal substrates of the microprocessor so they rely on neighboring canonical pri-miRNAs (Fang and Bartel, 2020; Shang et al., 2020).

The pre-miRNA is transported to the cytoplasm via exportin V where it undergoes the second cleavage reaction to form the mature miRNA/miRNA* duplex, which consists of the guide and passenger (*) strand (reviewed in Duchaine and Fabian, 2019; Loffreda et al., 2015). Dicer functions in miRNA maturation and helps to facilitate loading of the mature miRNA onto Argonaute (Foulkes et al., 2014). Dicer cleaves the terminal loop from the pre-miRNA stem creating the mature duplex that is 18–21 base pairs long and contains 3′ overhangs (Murphy et al., 2008). Dicer contains 2 catalytic RNAse III domains, C-terminal dsRBD, ATPase/RNA helicase, Piwi-Argonaute-Zwille (PAZ) domain, and the DUF283 domain (Foulkes et al., 2014; Gebauer et al., 2021). The PAZ domain recognizes the 5′ terminal phosphate of authentic pre-miRNAs and acts as a ruler to measure the cleavage site at the 3′ overhang where the RNAse III domains will cut a strand to create the mature miRNA duplex (Connerty et al., 2015). The mature miRNA duplex associates with the Ago protein to form the minimal effector miRNA-induced silencing complex (miRISC). The guide strand from the duplex becomes associated with the Ago protein and is unwound while the passenger strand is lost. This process is referred to as differential strand retention, which is based on the thermodynamic stability of the ends of the duplex (Bartel, 2018). The miRNA biogenesis involving the microprocessor is the canonical pathway and is depicted in Figure 1A. Research has shown that there are microprocessor-independent, or non-canonical, pathways for miRNA biogenesis (Miyoshi et al., 2010; Connerty et al., 2015; Bartel, 2018). Probably the most well-defined alternative miRNA biogenesis pathway is the one which combines intron splicing with dicing of the miRNA. These miRNAs are known as “mirtrons” and they are processed from RNAs that are both pre-miRNAs and introns (Westholm and Lai, 2011). Upon splicing of introns from transcribed RNAs, the processing of pri-miRNAs and pre-miRNAs is preceded by linearization of intron lariat by debranching enzymes. While it was originally thought that mirtrons are *Drosophila* and *C. elegans* specific pathways further studies found mirtrons throughout the animal kingdom (Siomi and Siomi, 2010; Salim et al., 2021). There are also miRNAs that are dicer-independent, but rely on the nuclear canonical machinery for miRNA biogenesis usually through cluster assistance, which will be discussed in detail in the next section (Fang and Bartel, 2020; Shang et al., 2020).

While this review focuses on miRNAs in the context of mammalian cells, it is important to note that miRNA biogenesis in plants is very similar to mammals, however, there are some key differences that should be highlighted. Similar to animal miRNA biogenesis, pri-miRNAs are transcribed in the nucleus by RNA polymerase II, followed by stabilization in a region known as the D-body (Voinnet, 2009; Wang et al., 2019). While in the D-body region the pri-miRNA interacts with a complex composed of zinc finger protein serrate (SE), a double-stranded binding protein hyponastic leaves 1 (Hyl1), dicer like 1 (Dcl1), and other accessory proteins depending on the specific type of miRNA (Wang et al., 2019). Dc11 functions much like Dicer in animal miRNA biogenesis, dc11 performs two cleavage reactions on the pri-miRNA; the first creating the shorter pre-miRNA, which then proceeds to another round of processing by dc11 to form the mature miRNA/miRNA* duplex. The mature duplex is exported out of the nucleus through HASTY (homolog to the animal exportin-V) (Bartel., 2004; Voinnet, 2009). Once in the cytoplasm, the guide strand interacts with Ago to guide the RISC to its target mRNA through near-perfect complementarity to silence the gene through direct cleavage and/or translational inhibition.

## RBPs in Control of miRNA Biogenesis

As the proper expression of miRNAs is essential for maintaining cellular homeostasis, their biogenesis is highly regulated at all steps, as highlighted in Figure 1B (Treiber et al., 2017; Nussbacher and Yeo, 2018). Analyses of crosslinking immunoprecipitation (CLIP) data with proteins involved in the miRNA biogenesis has helped identify specific RBPs involved in miRNA processing (Ramanathan et al., 2019). Post-transcriptional modifications of the RNA, as well as post-translational control of the biogenesis machinery and effector proteins involved in these processes, can impact the production of miRNAs and formation of the miRISC. Multiple modifications take place on miRNAs and the majority of them are important for their biogenesis, function, and stability; in addition, ubiquitination and phosphorylation of Ago proteins can play a role in miRISC formation (Peters and Meister, 2007; Meister, 2013; Müller et al., 2020; Wu et al., 2020). Some modifications have been extensively studied, including uridylation, editing of adenosine to inosine, or methylation of miRNAs, but many of them still need to be functionally characterized (Wyman et al., 2011; Michlewski and Cáceres, 2019). As an example, uridylation of pre-let7a miRNA by TUT4 or TUT7 blocks its processing and marks the miRNA for degradation (Heo et al., 2012; Michlewski and Cáceres, 2019). Deamination of specific adenosine to inosine by ADAR in pri-miR-142 (Yang et al., 2006) and pri-miR-151 (Kawahara et al., 2007) targets these miRNAs for degradation or blocks their processing, respectively. It was also shown that N6-methyladenosine (m6A) methylation by both mammalian or plant METTL3 homolog affects proper levels of mature microRNA (miRNA) biogenesis in human tissue cultures (Alarcón et al., 2015) and *Arabidopsis* (Bhat et al., 2020). The proposed mechanism involves specific methylation of a set of pri-miRNAs affecting proper folding of the RNA and recruitment of the microprocessor for proper and efficient pri-miRNA processing. In this case it’s the loss of the modification (m6A) in pri-miRNA that leads to the reduction of pri-miRNA-microprocessor interactions and reduction in the levels of mature miRNAs in both mammalian and plant studies. Additionally, experiments with an introduction of m6A marks by *in vitro* transcription in pri-miRNAs indicated more efficient processing of the modified pri-miRNA by the microprocessor, thus confirming the role of m6a modifications in miRNA biogenesis (Alarcón et al., 2015). Certain RBPs have been shown to increase or decrease efficiency of miRNA biogenesis through direct binding to the miRNA precursor and/or altering the machinery involved in the biogenesis. RBPs can influence pre-miRNAs through directly binding to the miRNA sequence or indirectly through binding to DICER and impacting its expression and/or function (Bicker et al., 2013; Connerty et al., 2015; Loffreda et al., 2015). PACT and TRBP are two RBPs that have been shown to bind to and stabilize the expression of DICER, thus indirectly increasing the fidelity of miRNA biogenesis (Peters and Meister, 2007; Ha and Kim, 2014). At the pri-miRNA level, RBPs have been shown to bind to the miRNA’s terminal loop. hnRNPA1 can bind to the stem loop of the pri-miR-18a (Díaz-Muñoz and Turner, 2018; Kooshapur et al., 2018). This binding alters the secondary structure, creating a more relaxed loop conformation which provides more accessibility for the microprocessor. On the other hand, RBPs can bind to the pri-miRNA and inhibit the biogenesis reaction through blocking the binding site for the microprocessor or other RBPs that are necessary for miRNA biogenesis (Loffreda et al., 2015; Michlewski and Cáceles, 2019). As depicted in Figure 1B the production of miRNAs is highly regulated by RBPs at multiple levels. As shown in Figure 1B, the two cleavage reactions that take place during miRNA biogenesis require the assistance of multiple RNA-binding proteins (RBPs). Fused in Sarcoma (FUS) protein is a ubiquitously expressed RBP that has been shown to directly interact with the microprocessor and recruit it to the transcription site of the miRNA (Morlando et al., 2012). Besides the FUS protein, a TAR DNA-binding protein-43 (TDP-43) has been shown to interact with both the microprocessor and Dicer complexes promoting the biogenesis of specific subsets of miRNAs and playing a role in neuronal differentiation (Kawahara and Mieda-Sato, 2012; Di Carlo et al., 2013). The RBP Ewing Sarcoma Protein (EWS) can inhibit the expression of DROSHA, likely through direct binding to the promoter (Ouyang et al., 2017), and thus regulate the overall efficiency of miRNA biogenesis. Another RBP that has been shown to interact with Drosha to influence miRNA biogenesis is SRSF3, a serine arginine splicing factor. SRSF3 regulates a large portion of canonical miRNAs (Kim et al., 2018). The CNNC motif lies about 17 nucleotides from the microprocessor and has been shown to interact with SRSF3 to aid in stimulating the processing of pri-miRNAs. It is thought that SRSF3 binding to the CNNC motif helps Drosha bind to the basal junction, thus aiding in pri-miRNA processing (Kim et al., 2018). Treiber and others performed a systematic analysis of pre-, pri- and mature miRNAs and identified a set of 72 human pre-miRNAs and found that 180 RBPs had preferential binding to a single or multiple miRNA precursor (Treiber et al., 2017). While it’s clear RBPs play a critical role in the biogenesis of miRNAs, most of these studies have been performed *in vitro* which could result in unphysiological binding. It will be important to investigate these interactions of RBPs with miRNA biogenesis pathways *in vivo* to identify their physiological significance as well as connect this type of regulation to cell type specific biogenesis of miRNAs.

There are multiple miRNAs that are made from pri-miRNAs that contain multiple clustered stem loop structures that were originally thought to be treated as independent units and thus individually cleaved by the microprocessor. However, it is becoming clear that certain pri-miRNAs that are poor substrates of the microprocessor are dependent on a close pri-miRNA neighbor for proper miRNA biogenesis, this process is known as cluster assistance. miR-451 is a miRNA that is dicer-independent but requires the canonical nuclear miRNA processing for its biogenesis as indicated with decreased miR-451 expression upon knockout of Drosha (Shang et al., 2020). miR-451 is a poor substrate for the microprocessor due to its short step loop, however, despite this, miR-451 is somehow a substrate of the microprocessor. Interestingly, miR-451 has been found to be tightly clustered by miR-144 throughout evolution (Fang and Bartel., 2020; Shang et al., 2020). miR-144 is an optimal substrate of the microprocessor and several groups have demonstrated that miR-451 biogenesis is dependent on its close proximity to miR-144. Shang and colleauges replaced miR-144 with miR-7a and miR-454, which are optimal substrates of the microprocessor, and miR-451 biogenesis was still promoted. This suggests a general mechanism whereby miRNAs that are suboptimal substrates of the microprocessor require canonical miRNAs in close proximity for their biogenesis (Shang et al., 2020). Additionally, several groups are identifying RBPs that are shown to assist in this clustering process (Fang and Bartel, 2020; Hutter et al., 2020; Kwon et al., 2020). Enhancer of rudimentary homolog (ERH) is a recently discovered component of the microprocessor that has been shown to aid in the cluster assistance process of miR-451 and miR-144 (Fang and Bartel., 2020; Hutter et al., 2020; Kwon et al., 2020). During cluster assistance the microprocessor is loaded onto the poor substrate with the aid of its neighboring high affinity miRNA substrate and ERH increases the processing of the suboptimal miRNA substrate through binding to the N-terminus of DGCR8 (Kwon et al., 2020).

Using a CRISPR/Cas9 LOF screen Hutter and colleuges found ERH and SAFB2 to be critical factors for cluster-mediated assistance (Hutter et al., 2020). Scaffold attachment factor B2 (SAFB2) is an RBP that has been found to be an accessory protein of the microprocessor in mammals (Hutter et al., 2020). The study by Hutter et al. looked at the miR-15a-16-1 cluster. Due to a large unpaired region in its basal stem, miR-15a is a suboptimal substrate of the microprocessor and therefore cannot be efficiently processed without the assistance from an optimal miRNA neighbor. Through use of a CRISPR/Cas9 screen they were able to identify SAFB2 as an essential cofactor for the efficient cleavage of pri-miR-15a through miR-16-1 assistance. Cluster assistance has also been observed in plants. In *Arabidopsis*, MAC5 is a component of the MOS4-associated complex which is needed for immunity and development (Palma et al., 2007; Li et al., 2020). MAC5 is an RBP that binds to stem loops and appears to help with cluster assistance. MAC5 is essential for plant development, as loss of function mutants of MAC5a and MAC5b have been shown to be embryonic lethal. Recently, MAC5 was shown to interact with the SE to promote pri-miRNA processing in plants via protecting the pri-miRNA from SE-dependent exoribonuclease activity (Li et al., 2020). Interestingly there is a human counterpart to MAC5, but it remains to be tested whether or not it interacts with the microprocessor in humans.

## miRISC Function and Nature of the miRISC

As mentioned above, once the miRNA combines with the Ago protein, the functional miRISC is formed. The Ago protein is the minimal effector needed for the miRNA to carry out its silencing mechanism (Connerty et al., 2015). Ago is made up of 4 domains; the N-terminal domain, Piwi-Argonaute-Zwille (PAZ) domain, the middle domain (MID), and the p-element induced wimpy testis (PIWI) domain (Song et al., 2004; Carthew and Sontheimer, 2009; Djuranovic et al., 2011; Sheu-Gruttadauria and MacRae, 2017). The N-terminal domain facilitates small RNA loading and unwinding of the duplex. The PAZ domain recognizes and anchors to the 3′ ends of miRNA. The MID domain binds the 5′ terminal monophosphate moiety and the 5′ terminal nucleotide of the miRNA-guide strand. Finally, the PIWI domain shows extensive homology to RNase H. Of note, it is important to mention that mammalian genomes encode 4 Ago proteins, Ago one to four, with Ago2 being most highly expressed and the only one to have endonucleolytic activity allowing it to cleave target mRNAs with full complementarity to miRNAs (Bhattacharyya et al., 2006; Gebert and MacRae, 2019; Kakumani et al., 2021). Plants and *C. elegans* have multiple Ago proteins that are further specialized in their cellular function and association with particular miRNA/small RNAs based on their length or 5′ nucleotide (Lim et al., 2003; Wang et al., 2019). The structure and function of Ago proteins have been well characterized (Hutvagner and Simard, 2008; Meister, 2013; Müller et al., 2020; Wu et al., 2020). The miRISC functions to identify the target transcripts first and then silence the gene expression of the target mRNA. The miRISC uses imperfect base complementarity to identify the target sequence in the mRNA. The target sites for the miRNA are usually located in the 3′UTR of the mRNA. Target site prediction is strengthened based on complementarity to the seed region. The seed region of the miRNA is from nucleotide 2 to 7 from the 5′ end, and its complementarity is one of the main criteria for target-site prediction (Bartel, 2009).

**FIGURE 2:**
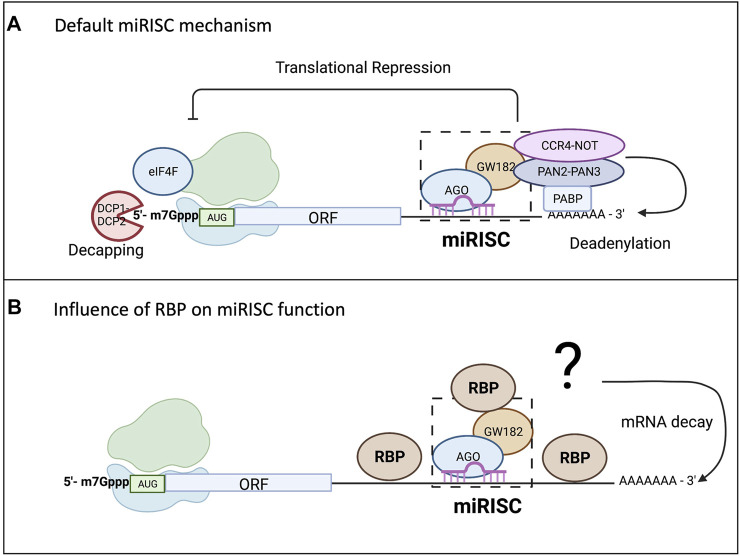
miRISC-mediated gene repression and the influence from RBPs. While there are conflicting models of miRISC-mediated gene silencing that are not mutually exclusive, scientists have agreed upon a “default” mechanism as all the proposed mechanisms for miRNA-mediated repression involve repression of translation and mRNA decay. As shown in **(A)**, Ago interacts with the PABP complex to promote mRNA deadenylation through recruitment of poly(A) nuclease deadenylation complex subunit 2 (PAN2)-PAN3 and carbon catabolite repressor protein 4 (CCR4)-NOT. Deadenylation promotes decapping by the mRNA-decapping enzyme subunit DCP1-DCP2, making the mRNA vulnerable to degradation by exoribonuclease 1 (XRN1). **(B)** Highlights the influence RBP binding in the 3′UTR can have on miRISC-mediated gene repression. The RBP can bind up or downstream of the miRISC and either enhance the repression, usually through increasing mRNA degradation, or it could reduce the silencing efficiency of the miRISC.

Unlike siRNA, miRNAs have imperfect base pair complementarity making target mRNA cleavage a rare event for mammalian miRNAs (Gebauer et al., 2021). miRNAs employ their silencing function mostly through translational repression and mRNA decay (Braun et al., 2012; Djuranovic et al., 2011; Fabian and Sonenberg, 2012; Bartel, 2018). While the exact mechanism of silencing remains a topic of debate, the “default” mechanism agreed upon includes inhibition of translation, followed by deadenylation, decapping, and decay of the mRNA transcript, as shown in Figure 2A (Djuranovic et al., 2011; Braun et al., 2013; Gardiner et al., 2015; Nawalpuri et al., 2020). Even though research has shown translational repression occurs first and might be one mode of controlling gene expression, it is the mRNA decay that ultimately consolidates and silences the target mRNAs (Bazzini et al., 2012; Bêthune et al., 2012; Djuranovic et al., 2011; Hu and Coller, 2012). However, it is important to note that this model of miRISC function will vary depending on additional factors as depicted in Figure 2B. There are several proposed models for miRISC-mediated translational repression, and they are not mutually exclusive (Gu and Kay, 2010; Fabian and Sonenberg, 2012). While the mature miRNA and Ago protein are part of the minimal miRISC, this association alone is insufficient to carry out miRISC-mediated translational repression. The more complete miRISC, including GW182, or its mammalian homologs TNRC6, is needed for translational repression (Eulalio et al., 2009; Huntzinger et al., 2013). GW182 is also a bridging factor between Ago proteins and the poly(A) binding protein (PABP) (Duchaine and Fabian, 2019), binding both of these proteins through the N-terminal domain. However, it is the carboxy terminal domain of GW182, which is referred to as the silencing domain, that recruits effector proteins such as deadenylases (PAN2-PAN3 and CCR4-NOT) and mRNA decapping factors (DCP1/2) (Eulalio et al., 2009; Braun et al., 2012; Wilczynska and Bushell, 2015). In a sequential series of actions, the miRISC is thought to induce translational repression of targeted mRNAs, which are later deadenylated by action of deadenylases, decapped by DCP1/2, and then degraded by the exosome and XRN1 (Rehwinkel et al., 2005; Braun et al., 2012). The presence of PABP aids in regulating both mRNA translation and mRNA turnover since it enhances miRNA-mediated deadenylation (Roy and Jacobson, 2013; Wigington et al., 2014). However, the presence of PABP on targeted mRNAs or its interaction with the miRISC is not necessary for translational repression (Djuranovic et al., 2011). Recently, 4EHP and GIGYF2 have been shown to bind to a conserved proline rich region of GW182 and impact miRISC-induced translational repression potentially at the step of 5′ mRNA cap binding (Chapat et al., 2017; Schopp et al., 2017). Regardless of whether there are multiple modes or one unifying model of translational repression by miRISC, it is becoming clearer that miRISC composition may influence outcome of the miRISC-target mRNA interaction. Both Ago and GW182 proteins may serve as molecular hubs for multiple ribonucleoprotein complexes (RNPs) or other enzymes involved in RNA metabolism further define their role in gene expression control.

## RBPs and a Functional miRISC

Multiple RBPs fulfill their gene regulation function independently of the miRISC but may impact the actions of the miRISC on target mRNAs. These RBPs can directly influence the functional outcome of the miRISC by modulating the actual composition of the miRISC or indirectly through interactions with the translational machinery involved in miRISC regulation. As previously mentioned, the FUS protein is an RBP that has been shown to directly associate with the core miRISC and influence the downstream function (Zhang et al., 2021). It is proposed that FUS facilitates the association between miRISC components such as the Ago protein, a set of mature miRNAs, and interacting with their mRNA targets, thus increasing efficiency of miRNA-mediated silencing. Since the interaction of FUS with the Ago protein is miRNA-independent, it may impact global miRNA regulation. FUS-enabled and selective miRISC targeting through direct interactions with “preferred” miRNAs and mRNA targets could create an even bigger challenge for “non-preferred” miRISC complexes and their ability to locate targets among the other RNAs in the cell.

In a very similar fashion, Smaug, an important RBP in the early development of *Drosophila,* has been shown to recruit the minimal miRISC complex or Ago proteins, regardless of the miRNA targeting (Pinder and Smibert, 2013). Smaug interaction with the miRISC or Ago proteins is driven by a direct protein-protein interaction (Smaug-Ago1/2) and recruitment of such a complex to the 3′UTR of targeted mRNAs is purely driven by Smaug-recognition elements (SREs). As such, the *Drosophila* Smaug-Ago1 complex does not require miRNAs for translational repression of Nanos mRNA. This direct recruitment of miRISC without the involvement of miRNA-mRNA target recognition has not been found in other cases, but several members of the Hu family of RBPs have been found to reduce miRISC function by preventing the formation of the repressive miRISC on target mRNAs or directly competing for components of translation machinery targeted by the miRISC (Fukao et al., 2015). The HuD member of the Hu family is known to stimulate eIF4A activity on bound mRNAs and thus prevent potential miRISC translational repression through the translation initiation scanning mechanism (Fukao et al., 2015). The ability of other members of Hu family, as well as other AU-rich element-binding proteins (ARE-BPs) to oligomerize on target mRNAs can also abrogate miRISC-mRNA interaction (as discussed in the next section).

There are multiple reports that the miRISC is indeed not a homogenous complex. The factors that make miRISC variations are associated with cell types as well as cellular processes such as cellular stress, growth, and differentiation. The heterogeneity of the miRISC complex allows for diversity in its function (Sheu-Gruttadauria and MacRae, 2017; Dallaire et al., 2018; Nawalpuri et al., 2020). The different cell-specific cofactors will contribute to the miRISC function, potentially changing the function of the same miRNA-Ago complex depending on the cell type. A recent study looked at miRNAs in somatic and germline cells and found that they formed distinct miRISC depending on the cell type (Dallaire et al., 2018). They used an *in vivo* fluorescent reporter with binding sites for miR-228 and germline- and somatic-specific promoters. They observed that in intestinal cells the miR-228 reporter was repressed at both the protein and mRNA level. However, they discovered stabilization of the miR-228 reporter in germline cells, suggesting a different mechanism where translational repression is uncoupled from mRNA destabilization. Using RNA affinity assays to purify specific miRISCs they identified a GW182-independent silencing mechanism used in germline cells of *C. elegans* compared to somatic cells. As such a single mRNA can have flexible regulation that changes based on cellular and developmental context, RBP presence, and miRISC composition, adding another layer of complexity to miRISC-mediated mechanisms and differential target regulation.

## Crosstalk Between miRNAs and RBPs on Target mRNAs

**FIGURE 3:**
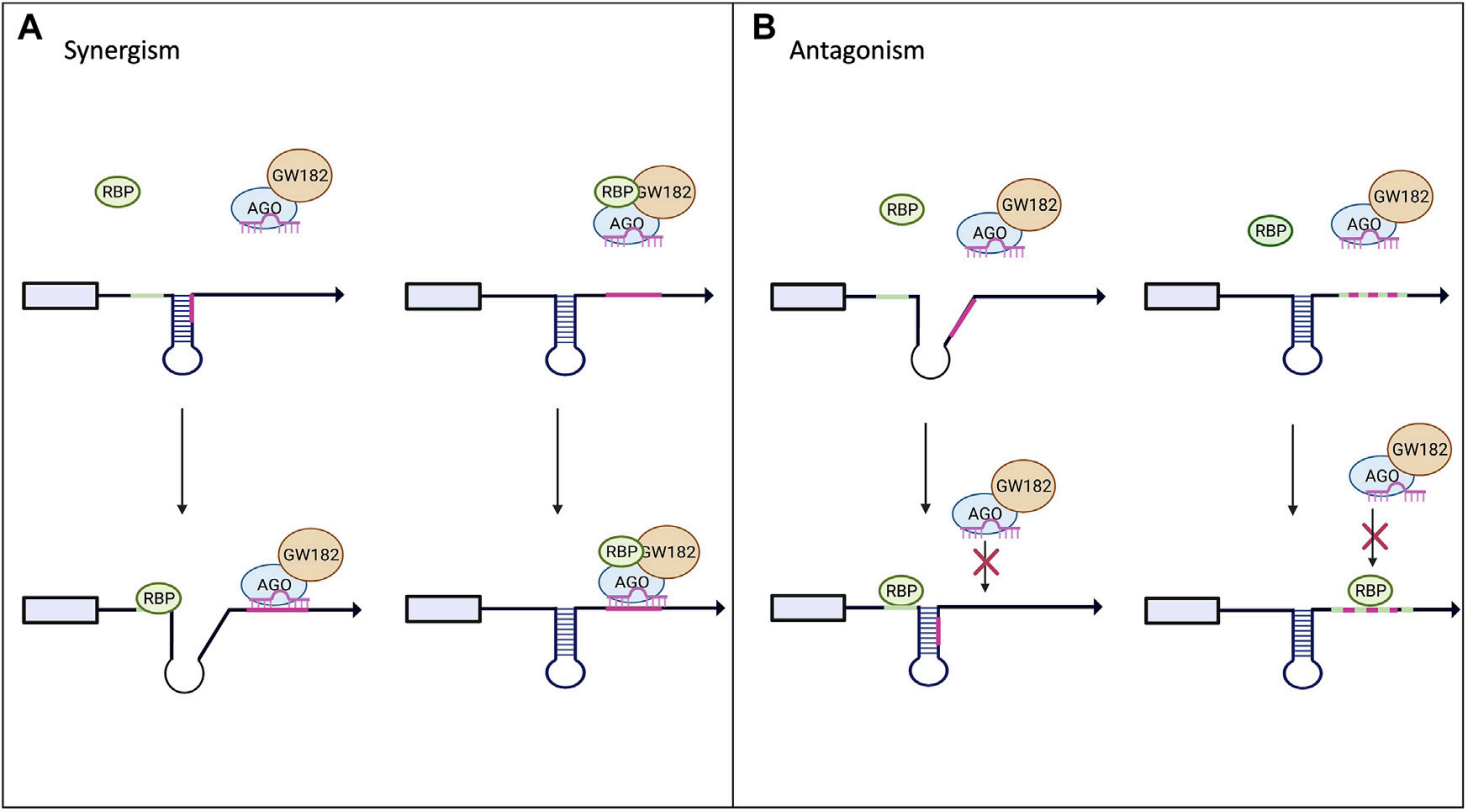
Interplay between RBPs and miRNAs on miRISC-mediated gene expression. Panel **(A)** shows potential models of synergism between RBPs and miRNAs. Upon binding to the mRNA target the RBP can alter the secondary structure and increase the exposure of the miRNA binding site allowing for increased miRISC binding. The RBP could also bind to the miRISC and increase its binding affinity to its target mRNA. **(B)** Highlights the possible antagonistic mechanism between RBPs and miRNAs. RBP binding to its target could alter the secondary structure, decreasing the miRISC’s access to its binding site. RBPs and miRNA can also compete for the same binding site and the RBP can outcompete and block the miRISC from binding to its site (Loffreda et al., 2015).

Historically miRNA research has focused on one individual miRNA, however, in an endogenous system there can be multiple miRNA binding sites in the 3′UTR of a single target mRNA. Currently miRbase has identified 1917 precursor and 2654 mature miRNAs. Additionally, over 1,000 RBPs have been identified in the human genome, so it is no surprise that their binding sites can be right next to each other, or even overlapping as depicted in Figures 3A,B (van Kouwenhove et al., 2011; Jiang et al., 2013; Cottrell et al., 2018). It has become apparent that in order to understand miRNA-mediated gene silencing fully, the miRNA must be studied in combination with other miRNAs and RBPs. Computational analysis has been helpful to predict crosstalk between RBPs and miRNAs (Jiang et al., 2013; Loffreda et al., 2015). PAR-CLIP and RIP-Seq experiments have been critical for identifying enrichment of RBP binding sites next to or overlapping with miRNA recognition target sites (Jiang and Coller, 2012; Iadevaia and Gerber, 2015; Ramanathan et al., 2019).

RBPs can have an antagonistic effect on miRISC gene silencing when competing for the same or nearby binding site within the 3′UTR of the target mRNA as demonstrated in Figure 3B. The best-known examples for this are Hu and ARE-BP family members (Jiang and Coller, 2012). Multiple groups have found that the CAT1 mRNA can be targeted by miR-122 and HuR (Filipowicz et al., 2008; Fabian and Sonenberg, 2012; Kundu et al., 2012). Under normal conditions, miR-122 targets CAT1 and represses its expression. However, it has been shown that when stimulated with stress, HuR can rescue CAT1 from miRNA-mediated repression by miR-122 (Filipowicz et al., 2008; Kundu et al., 2012). The mechanism by which HuR rescues CAT1 expression remains unclear; it could be through dissociation of miRNPs from the mRNA or prevention of the miRISC from binding to its target site in the 3′UTR. Another example of competition between miRNAs and RBPs is with HuR and miR-16. They both have binding sites in the 3′UTR of prostaglandin synthase cyclooxtgenesis-2 (COX-2). miR-16 normally binds to the 3′UTR of COX-2 and promotes rapid repression and degradation of the transcript. However, when HuR levels are increased, HuR can outcompete miR-16 for the binding site and stabilize COX2, thus increasing its expression (Young et al., 2012). Similarly, AU-rich element-binding protein 1 (AUF1) binds to AU-rich elements (AREs) in the 3′UTR of target mRNAs and potentially oligomerizes, preventing or enabling miRISC binding (Wilson et al., 1999; Zucconi et al., 2010). However, scientists have shown that AUF1 also has a high affinity for the let-7b miRNA. When AUF1 binds to the miRNA, let-7b, it facilitates its transfer onto Ago2, thus enhancing the miRNA-mediated repression (Yoon et al., 2014) in a way similar to FUS.

On the other hand, RBPs can cooperate with miRNAs to enhance the silencing of the target mRNA as shown in Figure 3A. This can be done through recruitment of the miRISC to the binding site on the target mRNA (example of Smaug and FUS above) or the binding of the RBP can change the secondary structure of the mRNA to increase the binding accessibility for the miRISC. Pumilio, a member of the Puf family, is an RBP that has been shown to enhance miRISC activity through unwinding the mRNA 3′UTR, thus promoting miRISC binding (Kedde et al., 2010; Miles et al., 2012; Nawalpuri et al., 2020). Pumilio is known to promote cell-cycle re-entry of quiescent cells upon binding to the 3′UTR of the mRNA that encodes for the tumor suppressor p27. Binding of Pumilo to the Pumilio-recognition elements (PREs) in the 3′UTR of p27 mRNA induces a change in the secondary structure of p27 mRNA that increases the accessibility of the target sites for miR-221 and miR-222, thus enabling the repression of this mRNA (Kedde et al., 2010). The similar mode of miRISC binding regulation and activity by Pumilio was found in a 3′UTR of transcription factors important for regulating cell proliferation, such as E2F3 (Miles et al., 2012).

While scientists have found examples showing antagonism and synergism between RBPs and miRNAs on miRISC gene silencing, it is important to note that all of these actions are co-occurring inside of the cell. Table 1 highlights certain known RBPs and miRISC interactions on common target mRNAs and you can see that a single RBP can either enhance or repress miRNA-mediated repression depending on the miRISC and RBPs binding sites. This suggests that there cannot be a universal model for the crosstalk between RBPs and miRNAs. A single mRNA can have binding sites for multiple RBPs and miRNAs, therefore the additive or countering effects of the different interactions will determine the final outcome of miRISC silencing efficiency.

**TABLE 1:** Known interactions of RNA-binding proteins and microRNAs.

Target	RNA-binding protein	microRNA	Action	References
Antagonistic interactions				
Transcription sites of miRNA	FUS	miR-9, miR-125b, miR-132	Drosha recruitment lost	Morlando et al. (2012)
CAT1 3′UTR	HuR	miR-122	HuR prevents miRISC from binding to target	Fabian and Sonenberg (2012); Kundu et al. (2012)
COX-2 3′UTR	HuR	miR-16	Compete for binding site in 3′UTR	Young et al. (2012)
VEGFA 3′UTR	hnRNPL	miR-297, miR-299	Competes with miRNAs for binding to VEGFA in 3′UTR	Shih and Claffey, (1999)
βTrCP1 (coding region)	CRD-BP	miR-183	Compete for binding in 3′UTR	Elcheva et al. (2009)
Stretches of uridine in 3′UTR	Dnd1	miR-430	Dnd1 makes target site inaccessible	Kedde et al. (2010)
Synergistic interactions				
RhoB	HuR	miR-19	Binding of HuR to recruits loaded miRISC	Sun et al. (2010)
p27, E2F3	Pumilio	miR-221/222	Pumilio binding alters secondary structure, increasing binding accessibility for miRISC	Kedde et al. (2010)
		miR-503		Miles et al. (2012)
c-myc	HuR	let-7	HuR recruits loaded let-7 RISC	Kim et al. (2009)
TNF-α	TTP	miR-16	TTP interacts with Ago to increase miR-16 loading	Jing et al. (2005)
Ago	AUF1	let-7a	Increase let-7 loading onto Ago	Yoon et al. (2014)
pre-miR18a	hnRNP1	miR-18a	Increase binding accessibility for miRISC	Michlewski et al. (2008)
pre-let7	DDX5	let-7	Facilitate miRISC loading to let-7 precurser	van Kouwenhove et al. (2011)

## Positional-Dependent Interactions of RBPs and miRNAs on miRISC Silencing

Many studies have indicated the influence of RBPs on miRISC-mediated gene silencing, but as different cells have varying RBP expression profiles, the same miRNA may have a different silencing mechanism or the transcript may be under control of both miRNAs and RBPs, depending on the specific cell type (Jiang et al., 2013; Cottrell et al., 2018). Many groups have investigated combined effects of miRNAs and RBPs, but studies are usually limited to a single mRNA or reporter 3′UTRs. As such, it is hard to conclude whether the exact mechanism that causes the antagonistic or synergistic effect of RBPs and miRISC on gene repression in a single 3′UTR is applicable in global analyses of other regulated genes. The answer to this question may lie in the positions of the miRNA and RBP binding sites in relation to one another in the 3′UTR of the target mRNA as well as in cell types. In an endogenous system the RBP binding site can be close to or far away from the miRNA binding site.

Recently two groups have identified that proximity of the binding sites influences the cross talk between RBPs and miRNAs (Cottrell et al., 2018). These studies identified that the closer the RBP and miRNA binding sites were to one another on target mRNA, the larger the influence of RBPs on miRISC gene targeting. One study indicated that nearby RBP binding was associated with enhanced miRISC targeting, which could potentially mean an increase in gene silencing. The other study used a massively parallel reporter (MPRA) assay with an eGFP plasmid library containing synthetic or endogenously encoded 3′UTRs, covering all possible combinations of several repressive translation elements (let-7 miRNA binding sites, AREs, PREs and SREs). Authors discovered positional effects between miRNA binding sites and AREs. AREs positioned upstream of the miRNA binding site of let-7 caused an increase in miRISC-silencing efficiency, while a decrease in let-7 silencing was observed when the AREs were downstream of the let-7 binding site (Cottrell et al., 2018). This effect was not only specific for miRNA binding sites but it was also seen in the combination of Pumilio binding sites (PREs) and AREs.

Interestingly, a study that looked at the ARE-BP, HuR, found that this RBP was able to mediate de-repression of Cat1 mRNA from miRISC silencing by binding to AREs located next to miRNA binding sites (Kundu et al., 2012). Given that HuR and other ARE-BPs are known to oligomerize upon binding to their target sites the group sought to investigate if the multimerization of HuR contributed to its ability to rescue Cat1 from miRNA repression. They created several mutants of HuR and found that the mutants with compromised multimerization of HuR did not interfere with the miRISC activity arguing for a steric occlusion model as a potential mechanism (Kundu et al., 2012). Such a model suggests the positional effect and distance-dependence through directional oligomerization of the RBPs may play a role in ARE-dependent modulation of miRISC activity. However, not all RBPs are known to oligomerize so this would not explain the mechanism for all RBP-miRNA crosstalk. Another possibility is that the RNA structure within the 3′UTR alters which would change the availability of the binding sites for both the miRISC and RBP, as described in models for Pumilio and miRISC on p27 and E2F3 mRNAs (Kedde et al., 2010; Miles et al., 2012).

A study in the zebrafish model demonstrated the impact of the RNA structure and RBP-binding sequence motifs during the maternal-to-zygotic transition (MZT) (Beaudoin et al., 2018; Vejnar et al., 2019). They found that certain AREs, U-rich and C-rich motifs, and miR-430 activity are responsible for variation in gene expression seen during the development. They identified multiple sequence and RNA structural elements can have antagonistic effects on the same mRNAs. Combination of these elements on the same mRNAs such as stabilizing U-rich motifs and destabilizing miR-430 target sites leads to differential temporal or spatial regulation and creates specific patterns of gene expression. The transcripts would be stabilized by maternally provided poly(U)-binding proteins and then deadenylated and degraded later in development. These actions are carried by combinatorial effects of the miR-430 miRISC and ARE-BPs by a dose-dependent mechanism established through either maternally deposited or newly synthesized RBPs and RNPs. It is clear the outcome of the interaction between RBPs and miRNAs can vary depending on their position from one another, primarily the distance between the binding sites and the local mRNA structure, thus further enforcing the idea that there is no single mechanism of miRISC function due to the these variations in mRNA targets. Scientists will need to identify methods to predict how certain miRNAs and RBPs interact with one another and identify their impact on gene expression regulation and then validate these results experimentally in endogenous targets. Additionally, other factors will be also at play, such as mRNA modifications or alternative splicing, that influence the structure within the 3′UTR, which in turn would alter miRISC and/or RBPs activity.

## Disease-Associated States Resulting From Interactions Between RBPs and miRNAs

Upon their initial discovery, miRNAs were believed to play a role in just development, but as more miRNAs were uncovered, scientists recognized their roles were much more diverse (Bartel, 2018). miRNAs likely play a role in every single biological process, and their dysregulation is seen in many diseased states, such as cancer and neurodegenerative disorders. Timed and correct expression of miRNAs in specific cell types is critical as mRNAs that are not silenced through miRNA-mediated repression will be translated into proteins and may impact cell growth, proliferation, or differentiation. The above-mentioned interplay between RBPs and miRNAs on certain targeted mRNA transcripts can easily contribute to pathogenesis if the levels of RBPs and miRNAs are altered. Most cancer cells see a decrease in global miRNA levels, but individual miRNAs have been shown to increase and accumulate in cancers (Peng and Croce, 2016; Vos et al., 2019; Zhang et al., 2021). Such variation in miRNA levels can indeed lead to tumorigenesis. For example, if there were an increase in miRNA regulators that target tumor suppressors, then there would be a decrease in the tumor suppressor genes (Cheng et al., 2017). In the opposite scenario, an increase in oncogenes can occur if there is a decrease in levels of their regulatory miRNAs. While the importance of miRNAs in cancer is evident, their role in cancer progression should be studied in parallel with RBPs. RBPs may be one of the key factors in determining miRNA function and mutations or changes in the expression of RBPs can impair miRNA biogenesis and miRISC activity. For instance, DEAD-Box 5 (DDX5) and DDX17 RNA helicases are RBPs that help regulate the Drosha-mediated cleavage to produce pri-miRNA. Studies have shown that there is an increase in DDX5 and DDX17 expression levels in breast, cervix, colon, and prostate cancer (Connerty et al., 2015; Shen and Hung, 2015; Khan et al., 2019). Knockdown of both DDX5 and DDX17, in human cervical carcinoma cells suppressed cellular proliferation indicating their association with abnormal cell growth. On the other hand, overexpression of DDX5 caused a proliferation of keratinocytes (van Kouwenhove et al., 2011) thus confirming its role in tumor phenotypes of these cells. It is worth mentioning that DDX5 exerts helicase activity when in the cytoplasm, and it facilitates miRISC loading by unwinding the let-7 precursor duplex.

Another example is an increased expression of COX-2 gene as a hallmark of colorectal cancer (Asting et al., 2011; Young et al., 2012). Under normal physiological conditions, miR-16 degrades COX-2, but miR-16 is decreased by about two-fold in colorectal cancer cells, thus its likelihood of binding and regulating COX-2 mRNA is drastically decreased. HuR expression is increased in colorectal cancer cells and since HuR also binds in the 3′UTR of COX-2 mRNA, additional stabilization of this mRNA and overexpression of COX-2 protein is warranted through the multimerization of HuR, a mechanism described in Kundu et al. (2012).

As Dicer is a major modulator of miRNA biogenesis it comes to no surprise that its level is important. Low levels of Dicer have been identified in several cancers associated with a poor outcome, such as breast, endometrial, lung, and ovarian cancer (Foulkes et al., 2014). However, there is also an observed increase in Dicer1 levels in the metastatic lesions in prostate cancer (Foulkes et al., 2014). This inconsistency in Dicer1 levels led scientist to focus on the specific mutations in the DICER gene rather than the levels. Both germline and somatic mutations in the DICER gene have been found in various cancers (Foulkes et al., 2014). Dicer1 syndrome is a rare genetic condition where specific mutations in the dicer gene predispose the patient for hereditary cancers (Caroleo et al., 2020). Only a third of Dicer1 pathogenic variant carriers present neoplasms during their life, suggesting there may be multiple additive events needed to create the neoplasm (Foulkes et al.,. 2014; Stewart et al., 2019; Caroleo et al., 2020). As the type of mutation in DICER gene can vary, future work will be needed to uncover how the specific type of mutation will alter the role of Dicer in recognition, binding, and processing of pre-miRNAs in addition to identifying the other events that may increase the chances of neoplasms occurring.

Neuronal development is another process that requires the function of genes whose expression is highly dependent on miRNAs. The proper expression of miRNAs is necessary for normal neuronal development, as altered levels of miRNA are observed for numerous neurodegenerative diseases (Juźwik et al., 2019). As we mentioned above, the components of the miRISC determine the function and mechanism of the miRISC and disruptions to these components, which are often due to RBPs, are observed in multiple neurodegenerative disorders. For example, brain atrophy, neurodegeneration, and gliosis are observed when Dicer is depleted in certain regions of the brain (Júzwik et al., 2019). FUS is involved in several biological processes and mutations in this RBP are observed in various neurodegenerative diseases, such as Amyotrophic Lateral Sclerosis (ALS) and Fronto-Temporal Lobar Degeneration (FTLD) (Loffreda et al., 2015; Nakaya et al., 2013). FUS has been shown to enhance the processing of certain miRNA by binding to the terminal loop of the pre-miRNA, these include miR-9, miR-125, and miR-132 (Morlando et al., 2012) which are all known to play important roles in neuronal functions (Loffreda et al., 2015). Cyclin-dependent kinases regulate the cell cycle and can initiate cell death. Their misregulation is often observed in neurodegenerative diseases, such as ALS, Parkinson’s and Alzheimer’s (Nguyen et al., 2002). CDK4 and CDK6 are known targets of the miRNA, miR-663a. miR-663a promotes cellular senescence by decreasing CDK4 and CDK6 expression (Kinoshita et al., 2021). Recent reports have found increased levels of CDK4 and CDK6 in the blood of ALS patients and connected this observation with combinatorial miRNA and RBP regulation (Katerina et al., 2018; Li et al., 2020; Kinoshita et al., 2021). Authors indicate that hnRNPH is an RBP that has been shown to indirectly decrease the expression of miR-663a. hnRNPH binds to RP11-670E13.6, which is a long noncoding RNA that, upon activation from hnRNPH binds to miR-663a and prevents miR-663a from binding to and repressing CDK4 and CDK6 mRNAs (Li et al., 2020:; Kinoshita et al., 2021). Exactly how increased levels of CDK4 and CDK6 contribute to ALS remains unclear, but it is evident that the interplay of RBPs and miRNAs contributes to this observation.

While there is clear evidence that disruption of miRISC machinery is seen in many neurodegenerative diseases, there is still a lack in understanding whether these disruptions of machinery cause the diseased state, or perhaps the disruption is a side-effect from another mishap that caused the disease (Kinoshita et al., 2021). Similarly, as an increase in pre-miRNAs is observed in tumor cells compared to normal cells, and might be a cause for tumorigenesis or metastasis, it would seem useful to study how changes in RBP expression influences miRNA biogenesis in certain cell types and cancers. Such data would clearly be beneficial in disentangling the complicated interactions between miRISC and RBPs.

## Conclusion

miRNAs play an essential role in gene regulation as they control the expression of genes involved in nearly all biological processes. Dysregulation of miRISC-mediated gene silencing is prevalent in human diseases, especially in neurological disorders and cancers. The importance of miRNAs is evident, and it’s becoming clear that in order to understand the biogenesis and function of miRNAs fully, RBPs, developmental stage, and cell types must be considered. Translational repression, modulated by RBPs and miRISCs, has been shown to be reversible (Bhattacharyya et al., 2006) so it is important to consider such regulation in spatial-temporal relations. The reversibility of translational repression modulated by RBPs and miRISCs allows for flexible control of the expression of targeted genes in a wide pool of mRNAs in a timely manner. This is, as mentioned earlier, important in cases such as cellular stress, cellular growth, or proliferation as well as during cellular specification (Nawalpuri et al., 2020). In some cases, “interruptions” in translation induced by miRISC and RBPs translational control will be enough to buffer cellular stress or developmental transition states. In other cases, additional gene expression control of transcribed but “unwanted” mRNAs would be further enforced by the miRISCs associated with mRNA decay factors or by interaction with RBPs. Establishing these connections between RBPs and miRISC components will be important as they will determine mechanisms by which miRISC complexes and RBPs regulate gene expression in biological systems. These studies require the novel design of experimental setups and new biochemical, genetic, and bioinformatics methodologies. Previous methods used to study the mechanism of miRISC mediated gene silencing focused on a single miRNA or a single RBP, looking at either single reporters or multiple endogenous genes but without a good overview of miRISC and RBP interactions. Current and future methods focus on uncovering RBPs and miRNAs’ combinatorial mechanism during miRISC- or RPB-mediated gene regulation. RNA element selection assays (RESA) have been useful for selecting RNA elements based on their activity *in vivo,* followed by high throughput sequencing to measure their regulatory function (Yartseva et al., 2017). The use of massively parallel reporter assay libraries (MPRAs) will be critical to study the individual and combined effects of miRNAs and RBPs both *in vitro* and *in vivo.* As there are infinite combinations of miRNAs and RBPs, the use of computational analysis for predicting the interactions of RBPs and miRNAs, followed by experimental validation, will help uncover the mechanism of certain RBPs and novel RNPs. Deciphering this crosstalk between RBPs and the miRISC in development stages and disease will be critical to identify new therapeutics.
